# A survey on the implementation of clinical medication reviews in community pharmacies within a multidisciplinary setting

**DOI:** 10.1186/s12913-024-11013-z

**Published:** 2024-05-03

**Authors:** S. Hogervorst, M.C. Adriaanse, M. Vervloet, M. Teichert, J.J. Beckeringh, L. van Dijk, J.G. Hugtenburg

**Affiliations:** 1 Department of Health Sciences, Faculty of Science, Vrije Universiteit, Amsterdam, The Netherlands; 2 Amsterdam Public Health Research Institute, Amsterdam UMC, Location VUMC, De Boelelaan 1117, Amsterdam, 1081 HV The Netherlands; 3 Department of Pharmaceutical Care, Nivel, Institute for Health Services Research, Utrecht, The Netherlands; 4 Department of Clinical Pharmacy & Toxicology, Leiden University Medical Center, Leiden, The Netherlands; 5 Westwijk Pharmacy, Amstelveen, The Netherlands; 6 Faculty of Science and Engineering. PharmacoTherapy, Epidemiology and Economics — Groningen, Research Institute of Pharmacy, Groningen, The Netherlands; 7 Department of Clinical Pharmacology and Pharmacy, Amsterdam UMC, Location VUMC, De Boelelaan 1117, 1081HV, Amsterdam, The Netherlands

**Keywords:** Clinical medication review, Implementation, Community pharmacy, Consolidated framework for implementation research, Questionnaire

## Abstract

**Background:**

Polypharmacy is common in chronic medication users, which increases the risk of drug related problems. A suitable intervention is the clinical medication review (CMR) that was introduced in the Netherlands in 2012, but the effectiveness might be hindered by limited implementation in community pharmacies. Therefore our aim was to describe the current implementation of CMRs in Dutch community pharmacies and to identify barriers to the implementation.

**Methods:**

An online questionnaire was developed based on the Consolidated Framework for Implementation Research (CFIR) and consisted of 58 questions with open ended, multiple choice or Likert-scale answering options. It was sent out to all Dutch community pharmacies (*n* = 1,953) in January 2021. Descriptive statistics were used.

**Results:**

A total of 289 (14.8%) community pharmacies filled out the questionnaire. Most of the pharmacists agreed that a CMR has a positive effect on the quality of pharmacotherapy (91.3%) and on medication adherence (64.3%). Pharmacists structured CMRs according to available selection criteria or guidelines (92%). Pharmacists (90%) believed that jointly conducting a CMR with a general practitioner (GP) improved their mutual relationship, whereas 21% believed it improved the relationship with a medical specialist. Lack of time was reported by 43% of pharmacists and 80% (fully) agreed conducting CMRs with a medical specialist was complicated. Most pharmacists indicated that pharmacy technicians can assist in performing CMRs, but they rarely do in practice.

**Conclusions:**

Lack of time and suboptimal collaboration with medical specialists are the most important barriers to the implementation of CMRs.

**Supplementary Information:**

The online version contains supplementary material available at 10.1186/s12913-024-11013-z.

## Background

As much as 48% of all people are using chronic medication in the European Union [1]. Taking five or more medications simultaneously is known as ‘polypharmacy’ and increases with age [2]. For the age group of 65–74, 25.3% are patients with polypharmacy, and this number increases to 46.5% for patients aged 85 years or older [3]. Polypharmacy can lead to the suboptimal use of medication and increases the likelihood of drug-related problems [4, 5]. For example, taking more drugs simultaneously increases the likelihood that one of these drugs leads to drug-related problems such as side-effects. The risk of drug-related problems increases linearly with the number of prescribed medication and higher age [6]. Even though almost half of drug-related problems are potentially preventable, they still account for more than 15% of hospital admissions [7].

A suitable intervention to target potential problems related to polypharmacy and drug related problems is a clinical medication review (CMR). A CMR is a structured, critical examination of a patient’s medicines. Its objective is to reach an agreement with the patient about treatment, optimizing the impact of medicines, minimizing the number of DRPs and reducing drug waste. The details of a CMR have been described in the publication by Mast et al. (2015) [8]. . The CMR aims to prevent worse outcomes or complications due to the disease or the pharmacotherapeutic intervention itself. The CMR was introduced in the Dutch primary care setting with the Multidisciplinary Guideline Polypharmacy and consists of the steps pharmacotherapeutic anamnesis [1], pharmacotherapeutic analysis [2], determining treatment plan with GP or specialist [3], determining treatment plan with the patient [4] and follow-up [5, 9].

Several studies have shown positive effects of CMRs, for example on lowering the amount of drug related problems or blood pressure [10–13]. However, other studies show limited or no effects of CMRs on clinical outcomes [14, 15]. For example, Huiskes et al. (2017) showed in their systematic review that CMRs resulted in a decrease in the number of drug-related problems, but minimal effects on clinical outcomes and no effects on quality of life [14].

This lack of consistent findings on clinical outcomes for CMRs might be explained by the degree in which CMRs are implemented, as several studies have shown major barriers for the performance of CMRs by pharmacists. In a Swiss evaluation on the implementation of CMRs, the authors concluded that successful implementation was hindered by a lack of a strong local network of community pharmacists with physicians, an effective workflow management and a practice- and communications-focused training for pharmacists and their teams [16]. In a German study, authors concluded that Pharmacist-led CMRs were hindered by a lack of patients’ confidence in pharmacists’ expertise and facilitated by pharmacies’ digital records of the patients’ medications [17].

In the Netherlands, the setting of our study, the Multidisciplinary Guideline Polypharmacy integrated the CMR into usual care in 2012 [9]. Two years after the development of this guideline in the Netherlands, CMRs were considered sub-optimally implemented according to Bakker et al. (2017) [18]. Their survey showed that the guideline was used only by 26% of the healthcare providers (HCPs) involved and that only 43% of the patients with polypharmacy had their medication assessed in the year previous to the survey. Factors contributing to the lack of implementation were the large number of patients eligible for a CMR, inadequate selection criteria, the time consuming and inefficient review procedure, a lack of collaboration between HCPs and insufficient reimbursement [18]. In order to improve the implementation rate, in 2015 the Dutch Health Inspectorate (DHI) tightened the selection criteria and required community pharmacists (CPs) and general practitioners (GPs) to annually perform a minimum number of CMRs in high-risk patients only [19].

The current study expands on the study by Bakker et al. (2017) [18] and aims to give an update on the nationwide implementation of CMRs, ten years after the inception of the Dutch Multidisciplinary Guideline Polypharmacy. The aim of this study was to describe the current implementation of CMRs in Dutch community pharmacies and to identify barriers that might hinder the implementation.

## Methods

### Study design and setting

An online questionnaire on the implementation of CMRs was sent out by e-mail to all community pharmacies (*N* = 1953) in the Netherlands by making use of the membership list of the Royal Dutch Association of Pharmacists (Dutch: Koninklijke Nederlandse Maatschappij ter bevordering der Pharmacie (KNMP)).

### Ethics approval and consent to participate

In the Netherlands, completing a survey does not fall under the scope of the Dutch Medical Research Involving Human Subjects Act [32]. This law states that medical ethical review is only required when research involves human subjects and if people are being subjected to actions or if rules of behaviour are imposed on them. As our research consisted of a questionnaire that did not impose any rule or behaviour on our subjects, a medical ethics review of the study protocol was not required. Therefore, all methods were carried out in accordance with relevant guidelines and regulations.

### Questionnaire design

The questionnaire was developed by two researchers (SH and JH) based on the Consolidated Framework for Implementation Research (CFIR). The CFIR is an implementation framework aimed to evaluate an implementation study or to design an implementation study [20]. The CFIR provides a pragmatic structure for approaching complex, interacting, multi-level, and transient states of constructs in the real world by embracing, consolidating, and unifying key constructs from published implementation theories. The CFIR consists of the domains intervention characteristics, outer setting, inner setting, characteristics of individuals and process. Each domain is divided into several constructs. This study used the CFIR framework as a guide to ensure all relevant concepts for implementation were present in the developed questionnaire to promote the generalizability of this research. Some CFIR constructs were omitted because they were deemed not relevant for this study based on consensus discussions between SH and JH. For an overview of included and omitted CFIR constructs, see Table 1. Appendix A gives a full overview of the developed questionnaire and all of its questions according to CFIR domains and constructs. The final questionnaire consisted of 58 questions with either multiple choice, Likert-scale or open ended answering options.

**Table 1:** Overview of omitted CFIR domains in questionnaire development with both a brief description of the domain according to the CFIR [20] and reason for omittance in *italics*

Construct		included/reason for omittance
**I. INTERVENTION CHARACTERISTICS**		Included domains: Evidence Strength and Quality (B), Adaptability (D), Complexity (F), Cost (H).
A	Intervention Source	Perception of key stakeholders about whether the intervention is externally or internally developed. *Perception of stakeholders on the source of the intervention was not deemed relevant as clinical medication reviews are integrated into routine care and therefore pharmacists are obliged to conduct the intervention.*
C	Relative Advantage	Stakeholders’ perception of the advantage of implementing the intervention versus an alternative solution. *Relative advantage was not deemed relevant, as the national guideline polypharmacy requires pharmacists to carry out this service and no alternative solution is available.*
E	Trialability	The ability to test the intervention on a small scale in the organization, and to be able to reverse course (undo implementation) if warranted. *Trialability was not deemed relevant as the clinical medication review was already implemented into usual care ten years ago in the Netherlands.*
G	Design Quality & Packaging	Perceived excellence in how the intervention is bundled, presented, and assembled. *Design quality & packaging not deemed relevant as the intervention is a conversation without any physical materials.*
**II. OUTER SETTING**		Included domains: Patient Needs & Resources (A), Cosmopolitanism (B), External Policy & Incentives (D).
C	Peer Pressure	Mimetic or competitive pressure to implement an intervention; typically because most or other key peer or competing organizations have already implemented or are in a bid for a competitive edge. *Peer pressure to implement the intervention was not deemed relevant as the intervention is already integrated into usual care throughout the Netherlands by means of a clinical guideline 10 years ago.*
**III. INNER SETTING**		Included domains: Structural Characteristics (A), Networks & Communications (B), Goals & Feedback (D5), Available Resources (E2), Access to Knowledge and Information (E3).
C	Culture	Norms, values, and basic assumptions of a given organization. *Culture was omitted as it is a difficult domain to assess in a quantitative questionnaire. Moreover, the community pharmacist is often the owner of the pharmacy and therefore answers might be socially desirable.*
D	Implementation Climate	The absorptive capacity for change, shared receptivity of involved individuals to an intervention, and the extent to which use of that intervention will be rewarded, supported, and expected within their organization. *Implementation climate was omitted as it is a difficult domain to assess in a quantitative questionnaire. Moreover, the community pharmacist is often the owner of the pharmacy and therefore answers might be socially desirable.*
1	Tension for Change	The degree to which stakeholders perceive the current situation as intolerable or needing change. *Tension for change is not deemed relevant as the intervention is already integrated into usual care.*
2	Compatibility	The degree of tangible fit between meaning and values attached to the intervention by involved individuals, how those align with individuals’ own norms, values, and perceived risks and needs, and how the intervention fits with existing workflows and systems. *Compatibility was deemed a relevant domain, but no specific questions were included in this questionnaire. Instead, compatibility of the intervention can be assessed by analyzing implementation success with determinants such as pharmacy’s patient population, composition of staff or being part of specific chains or formula. However, this study aimed to describe the implementation, and no uniform value judgement on implementation success was available with which to analyze compatibility.*
3	Relative Priority	Individuals’ shared perception of the importance of the implementation within the organization. *Relative priority is not deemed relevant as the intervention is already implemented by means of a national guideline ten years ago.*
4	Organizational Incentives & Rewards	Extrinsic incentives such as goal-sharing awards, performance reviews, promotions, and raises in salary, and less tangible incentives such as increased stature or respect. *External incentives and rewards, such as reimbursement for CMRs by health insurers, fall under ‘Outer setting; External policy and incentives’. Organizational incentives and rewards were omitted because the questionnaire was filled out by the community pharmacist, and therefore we were unable to ask about increased stature or respect that individual pharmacy technicians might gain in their community pharmacy teams by conducting CMRs.*
6	Learning Climate	A climate in which: (a) leaders express their own fallibility and need for team members’ assistance and input; (b) team members feel that they are essential, valued, and knowledgeable partners in the change process; (c) individuals feel psychologically safe to try new methods; and (d) there is sufficient time and space for reflective thinking and evaluation. *Learning climate was omitted as it is a difficult domain to assess in a quantitative questionnaire. Moreover, the community pharmacist is often the owner of the pharmacy and therefore answers might be socially desirable.*
E	Readiness for Implementation	Tangible and immediate indicators of organizational commitment to its decision to implement an intervention. *Readiness for implementation is not deemed relevant as the intervention is already implemented.*
1	Leadership Engagement	Commitment, involvement, and accountability of leaders and managers with the implementation. *Leadership engagement was omitted as the community pharmacist filled out the questionnaire, so no questions regarding the engagement of leadership could be asked to any other pharmacy staff members.*
**IV. CHARACTERISTICS OF INDIVIDUALS**		Included domains: Knowledge & Beliefs about the Intervention (A), Self-Efficacy (B), Other Personal Attributes (E).
C	Individual Stage of Change	Characterization of the phase an individual is in, as he or she progresses toward skilled, enthusiastic, and sustained use of the intervention. *Individual stage of change was omitted as the intervention is integrated into usual care in the Netherlands 10 years ago.*
D	Individual Identification with Organization	A broad construct related to how individuals perceive the organization, and their relationship and degree of commitment with that organization. *Individual identification with organization was omitted as the community pharmacist filled out the questionnaire, so the view of other pharmacy staff members could not be surveyed.*
**V. PROCESS**		Included domains: Planning (A), Engaging (B), External Change Agents (B4), Executing (C), Reflecting & Evaluating (D)
1	Opinion Leaders	Individuals in an organization who have formal or informal influence on the attitudes and beliefs of their colleagues with respect to implementing the intervention. *Opinion leaders are not included in the questionnaire as the questionnaire was always filled out by the community pharmacists, which did not allow for distinction of roles within the community pharmacy.*
2	Formally Appointed Internal Implementation Leaders	Individuals from within the organization who have been formally appointed with responsibility for implementing an intervention as coordinator, project manager, team leader, or other similar role. *Internal implementation leaders are not included in the questionnaire as the questionnaire was always filled out by the community pharmacists, which did not allow for distinction of roles within the community pharmacy.*
3	Champions	“Individuals who dedicate themselves to supporting, marketing, and ‘driving through’ an [implementation]” [101] (p. 182), overcoming indifference or resistance that the intervention may provoke in an organization. *Champions are not included in the questionnaire as the questionnaire was always filled out by the community pharmacists, which did not allow for distinction of roles within the community pharmacy.*

Before sending the questionnaire to all community pharmacies in the Netherlands, the questionnaire was pilot tested for face validity by four community pharmacists, working in different types of pharmacies (independent, formula (i.e. franchise) and chain). The communication department of the Royal Dutch Association of Pharmacists reviewed the questionnaire before distribution. Both the pilot testing and review by the communication department did not lead to any major changes in the questionnaire, apart from changing the order in which questions were asked.

### Data collection

Data were collected by using the survey software Questback (SaaS, Questback, Oslo, Norway). Responses were collected from January 5th 2021 till January 18th 2021. A reminder was sent out on January 15th. 289 pharmacists responded to the questionnaire (response rate = 14.8%). Fourteen pharmacists were excluded from the analyses, because they worked in outpatient hospital pharmacies, pharmacies serving nursing homes and central filling pharmacies, and their patient population is generally different from the patient population of community pharmacies and they rarely conduct a CMR. Pharmacists received an invitation letter by mail before the start of the questionnaire. Informed consent was asked prior to the questionnaire.

### Analyses

All data were analyzed using SPSS 27.0 (IBM Corp., Chicago, USA). Descriptive statistics were used to provide percentages (categorical variables) or means and standard deviations (continuous variables). Open ended questions are presented as quotes.

## Results

### Characteristics of included pharmacies

Table 2 gives an overview of characteristics of the included pharmacies. A total of 275 pharmacists were included in analyses. Community pharmacies had 9,438 patients on average. Almost half (49.3%) of community pharmacies were located in a healthcare center and the majority (70.6%) were part of a pharmacy chain or formula.

**Table 2:** Characteristics of the included pharmacies (*N* = 275)

Number of fte pharmacists	Mean (sd) – range	1.44 (0.622)	1–4
Number of fte pharmacy technicians	*Mean (sd) – range*	6.21 (2.750)	1–27
Number of planned CMRs	*Mean (sd) – range*	86.9 (65.754)	0–800
Size of patient population	*Mean (sd) - range*	9438 (4192)	1000–25,000
Average demographic patient composition	*Migration background (non-western), %* *Migration background (western), %* *Non-migration background, %* *Other, %*	11.7 6.0 73.8 8.5	
Average socioeconomic patient status	*Low, %* *Average, %* *High, %*	25.2 62.8 12.1	
Pharmacy located in a healthcare center	*%*	49.3	
Pharmacy as part of a chain/formula^a^	*%*	70.6	

Fte, fulltime equivalent; CMR, clinical medication review; SD, standard deviation

^a^Pharmacies part of a chain or formula include: Acdapha, Alphega, BENU, Boots, Medsen, Pluriplus, Service Apotheken

### Intervention characteristics

Results are presented according to the five CFIR domains. Most of the pharmacists agreed that a CMR has a positive effect on the quality of pharmacotherapy (91.3%) and on medication adherence (64.3%). The majority of pharmacists (79.6%) considered conducting a CMR with patients of which a medical specialists was the prescribers as complex. Most pharmacists (95.6%) indicated that health insurers should use a uniform reimbursement for CMRs. Most common parameters used for selecting patients for a CMR included the number of medications in chronic use (92.0%), age (89.1%) and the specific GP by which a patient is treated (58.5%), followed by poor medication adherence (55.6%), cognition (49.8%), fall risk (49.1%) and residential location such as living in a nursing home (47.6%).

### Outer setting

Table 3 gives an overview of the opinion of pharmacists on statements related to the CFIR domain ‘outer setting’. Almost all pharmacists thought that a CMR promotes the relationship with patients (93.5%) and the GP (90.2%). Most pharmacists (73.9%) thought that the content of a CMR meets the patient’s care needs. Almost half of the pharmacist stated that they never or rarely met with homecare (42.2%) or a medical specialist (44.7%) to discuss a CMR. Patients were most often invited for a CMR by phone (66.1%), followed by letter (23.5%), by other means not specified (7.3%), at the front desk (2.4%) or by e-mail (0.7%).

**Table 3:** Descriptive statistics on community pharmacists’ responses to questions about the CFIR domain ‘outer setting’ regarding the implementation of CMRs (*N* = 275)

Patients or family members asked for a CMR out of own initiative	
Never/rarely, % Sometimes, % Often/always, %	21.8 75.1 3.1
A CMR promotes my personal treatment relation with the patient	
*Strongly disagree/disagree, %* *Neutral, %* *Agree/strongly agree, %*	0.7 5.8 **93.5**
Content of a CMR meets patient’s care needs	
*Never/rarely, %* *Sometimes, %* *Often/always, %*	1.0 25.1 **73.9**
Frequency of topics discussed with patients during a CMR (% of often & always)	
Adverse drug events, *%* *(long-term) effects of medication, %* *Not being able to take medication, %* *Forgetting to take medication, %* *Unwillingly skipping intake moments, %* *Costs of medication, %* *The amount of medication, %* *The availability of medication, %*	**79.6** 30.1 49.2 56.5 45.3 10.0 **65.0** 49.1
Frequency of consultations with a medical specialist about a CMR	
*Never/rarely, %* *Sometimes, %* *Often/always, %*	**44.7** 48.0 7.3
Frequency of consultations with homecare about a CMR	
*Never/rarely, %* *Sometimes, %* *Often/always, %*	**42.2** 37.1 20.7

CFIR, consolidated framework for implementation research; CMR, clinical medication review; GP, general practitioner

### Inner setting

Table 4 shows the answers of pharmacists regarding the CFIR domain ‘inner setting’. Pharmacists conducted 56 CMRs annually on average. Pharmacies participated on average annually in 2.8 pharmacotherapeutic audit meetings (PTAM, i.e. regular meeting between CPs and GPs about pharmacotherapy). 41.1% of pharmacists set their own additional goals for conducting CMRs. Most of these own goals relate to specific target populations, such as elderly patients or other vulnerable patient populations. The majority (90.0%) of pharmacists made use of a structured treatment plan when conducting a CMR.

**Table 4:** Descriptive statistics on community pharmacists’ responses to questions about the CFIR domain ‘inner setting’ regarding the implementation of CMRs (*N* = 275)

Number of conducted CMRs	Mean (SD) – range	56 (50.2)	0–300
Pharmacy sets its own additional goals for conducting CMRs	*Yes, %*	41.1	
Pharmacist asked an external party to conduct CMRs for them	*Yes, %*	6	
- Amount of CMRs conducted by external parties	* Mean (sd) - range*	97 (50.8)	15–170
System used to register CMRs	*Medicijnmonitor, % * *NControl, %* *Pharmacy tech, %* *Different, %*	8.3 31.8 29.4 30.4	
Average time to conduct a CMR pharmacotherapeutic analysis	*Average time (in minutes + SD) - range*	**56.2** (26.4)	10–240
Average time to conduct a CMR pharmacotherapeutic treatment plan	*Average time (in minutes + SD) - range*	12.2 (8.4)	5–120
How often do you or a GP request lab research to inquire about patients clinical values as part of a CMR?	*Never/seldom, %* *Sometimes, %* *Often/always, %*	7.6 58.1 43.2	
Do you make use of a structured questionnaire during pharmacotherapeutic anamnesis?	*Never/seldom, %* *Sometimes, %* *Often/always, %*	3.8 6.2 **90.0**	
How is the local cooperation between the GP(s) and pharmacist legally organized regarding the conducting of a CMR?			
	*Regional agreement, %* *Agreement with the GP(s) with whom they share a large part of the patient population, %* *Agreement with the GP(s) from the PAM group, %* *Without any agreement, %* *Other, &*	7.6 19.6 32.4 30.5 9.9	

CFIR, consolidated framework for implementation research; CMR, Clinical medication review; PAM, Pharmacotherapeutic audit meetings (Dutch: Farmacotherapeutisch overleg); SD, standard deviation; GP, general practitioner

Table 5 shows the responses of community pharmacists on statements regarding the implementation of CMRs in community pharmacies related to the CFIR domain ‘outer setting’. Almost half (42.5%) of pharmacists indicated that they lack time to conduct a CMR and the majority of pharmacists (67.3%) considered the reimbursement for conducting a CMR insufficient. The START-/STOP criteria are used by almost all pharmacists (92.4%) as an aid in conducting CMRs. Other popular tools and criteria are the guideline database of the Dutch Association of Medical Specialists (85.1%), the STRIP method (69.8%), the NHG-standard (56.4%), the Beers criteria (39.6%) and Ephor (39.6%) [21–27].

**Table 5:** Descriptive statistics on community pharmacists’ responses to statements about the CFIR domain ‘inner setting’ regarding the implementation of CMRs (*N* = 275)

	Strongly disagree/disagree, %	Neutral, %	Strongly agree/agree, %
I do have enough time to conduct a complete CMR*	42.9	28.0	29.1
**The reimbursement that a pharmacy receives for a CMR is sufficient**	67.3	21.8	10.9
**Conducting a CMR promotes my professional relationship with a GP**	1.1	8.7	90.2
**Conducting a CMR promotes my professional relationship with the medical specialist**	33.5	45.1	21.4
There are sufficient local cooperation agreements to properly conduct CMRs*	29.4	16.7	53.9
Patients do want to participate in a CMR even though costs are deducted from the ‘own risk rate’*	48.4	27.6	24.0
**There is a suitable room in the pharmacy where a CMR can be conducted**	3.3	5.8	90.9
**The pharmacy information system supports the performance of a CMR sufficiently**	33.5	24.0	42.5
**The information system of different healthcare providers must be compatible to the pharmacy information system**	2.9	16.0	81.1

*Statement was reversed. The statement was surveyed negatively (e.g. ‘I do *not* have enough time…) but was made positive in order to increase the readability of the table

CFIR, consolidated framework for implementation research; CMR, clinical medication review; GP, general practitioner

### Process

Most pharmacists think a pharmacy technician (51.7%) or a higher vocationally educated pharmacy technician (67.3%) can assist the pharmacist in conducting a CMR. About half of the pharmacists considered the selection criteria for CMRs specific enough (52.0%) and (strongly) disagreed (55.7%) that there is a lack of a systematic approach to conduct a CMR. Almost half (48.1%) of pharmacists were stimulated to conduct more CMRs by external organizations, with an average surplus of 13.7 extra CMRs conducted as a result of external stimulation in 2020 per pharmacy. The most common external organization to stimulate a pharmacy to conduct more CMRs were pharmacy chains or formulas and were mostly related to the amount of CMRs conducted (49.0%). For the pharmacotherapeutic anamnesis, the most common method was that patients fills in the questionnaire together with a pharmacy staff member by phone (43.9%), followed by filled in together in the pharmacy (36.7%), filled in by the patient him/herself (17.2%) or together during a video call (2.2%).

Table 6 shows who is responsible for the different stages in the implementation of a CMR according to community pharmacists. In most of the cases, pharmacists and GPs share the responsibility of selecting patients. They also share the responsibility of setting up and evaluating the pharmacotherapeutic treatment plan. In approximately 10% of the pharmacies, pharmacy technicians are responsible for inviting patients and conducting the pharmacotherapeutical anamnesis. Also, in a quarter of pharmacies the GP is responsible for determining the treatment plan with the patient. If a pharmacist filled in ‘other’, GPs assistants were the most common answer. Lastly, in 6.5% of the pharmacies the follow-up and monitoring does not happen.

**Table 6:** Responsibilities in the different stages of the implementation process of a CMR

Responsibility	Pharmacist, %	Pharmacist and GP, %	GP, %	GP and medical specialist, %	Pharmacy technician, %	Higher vocationally educated pharmacy technician, %	Other, %	Doesn’t happen (yet), %
**selecting patients**	35.3	53.5	-	4.0	-	-	7.3	-
**inviting patients**	72.4	-	-	6.2	8.4	2.5	10.5	-
**the pharmacotherapeutic anamnesis**	82.2	-	-	0.4	6.9	2.2	8.4	-
**the pharmacotherapeutic analysis**	95.6	4.4	0.0	0.0	-	-	-	-
**the pharmacotherapeutic treatment plan**	40.7	51.6	6.2	-	-	-	-	-
**the follow-up and monitoring**	26.9	44.4	16.0	-	-	-	6.2	6.5
**the offering the treatment plan to the patient**	59.6	-	25.5	-	2.2	1.5	11.3	-

GP, General Practitioner; CMR, clinical medication review

-, answer option not included in the questionnaire

## Discussion

### Main findings

In this study the implementation of CMRs in community pharmacies was investigated with a structured online questionnaire based on the CIFR and identified barriers to the implementation of CMRs. Most of the pharmacists agreed that a CMR has a positive effect on the quality of pharmacotherapy and medication adherence. The results further show that pharmacists believe conducting CMRs improves their relationship with GPs and meets patient’s care needs. Collaboration with GPs is generally well established, but collaboration with medical specialists in CMRs is considered complex by the majority of pharmacists. Additionally, both home care and medical specialists are consulted in about half of all cases. The process of conducting CMR takes pharmacists an hour on average, and the majority of pharmacists indicated a lack of time as a major implementation barrier. Pharmacists have indicated that some aspects of conducting a CMR can be delegated to (higher vocationally educated) pharmacy technicians, but the majority of tasks is currently being done by Pharmacists and/or GPs.

### Comparison with existing knowledge

Implementing the CMR following the adoption of the guideline ‘Polypharmacy in the elderly’ in 2012, initially proved to be difficult [9, 18, 19]. It appeared that in 2014 only 26% of the community pharmacists performed CMRs [18]. The survey showed that the problems were related to the inadequate selection criteria by which a very large number of patients would be eligible for a CMR, the extraordinary amount of time it would take to perform a CMR in these patients (approximately two hours per patient), and the lack of appropriate remuneration for this specific care activity, which most HCPs did not consider being a part of usual care. In a recent international systematic review on the implementation of CMRs in community pharmacies, collaboration with doctors and insufficient remuneration were also the most dominant barriers [28]. Accordingly, it was concluded that the problems could best be addressed through stricter patient selection, the use of more efficient working methods and the availability of appropriate remuneration [18].

Ten years later, our study showed that the implementation has improved. By now a systematic approach is available and is considered sufficient by most pharmacists, which has decreased the time spend on a CMR by community pharmacists per patient to about an hour. Most pharmacists also do consider the selection criteria appropriate and different tools and guidelines are used more frequently. The majority of pharmacists now involve and consult with GPs on different sub-tasks of CMRs and consider collaborating with GPs as an improvement to the professional relationship they have with GPs. All of this indicates that most major barriers of the past have been overcome, but some other barriers persist. Collaboration with medical specialists is still considered to be complex by most pharmacists, and medical specialists and homecare organizations are not consulted about CMRs with their patients in all cases. Many patients with polypharmacy switch between primary and secondary care, and medication problems derived from collaboration between care domains is a persistent problem [29, 30]. Future studies should therefore explore different ways to improve the relationship with medical specialists and home care organizations. Future studies should therefore explore different types of medical specialists’ views on medication management and their preferred roles in CMRs.

Moreover, lack of time was a barrier identified both in the study by Bakker, Kemper, Wagner et al. [18] and in our results. Over the years, several attempts have been made to increase CMR efficiency and limit the time required to perform a CMR. Our results suggest that (higher vocationally schooled) pharmacy technicians can play a role in conducting some tasks of a CMR according to pharmacists, but rarely do in practice. A recent study on the implementation of CMRs in the German care setting also showed that pharmacists themselves believed that involving pharmacy technicians in some CMR tasks could improve the implementation of CMRs [31]. We therefore suggest delegating some tasks to (vocationally trained) pharmacy technicians. Especially in the first few steps of a CMR, such as the selection of patients and the gathering of important patient data during the pharmacotherapeutic anamnesis, technicians can limit the time spend by community pharmacists on CMRs.

Another potential method to increase efficiency for CMRs is to make more use of integrated information and communication technoclogy (ICT) systems, i.e. shared ICT systems between CP and GP teams. Although medication data and lab values of most patients have become available via a national electronic platform, the separate information systems of most pharmacies and general practices are not interconnected. Ideally, key clinical data needed for a CMR such as an overview of current medication, blood pressure or kidney function parameters as well as data generated in the course of a CMR should be readily available and shared between GP practices and pharmacies, observance to patients privacy.

### Strengths and limitations

The strengths of this study include the questionnaire sent out to all community pharmacies in the Netherlands by the Royal Dutch Association of Pharmacists (KNMP). The use of the CFIR framework as a basis for the questionnaire ensured that relevant aspects of implementation are included [20]. Using a questionnaire ensured that all pharmacists in the Netherlands could be approached by e-mail which increases the generalizability of the results.

This study reached a response rate of 14.8%. This response rate is too low to take our results as representative for the whole group of community pharmacists. Assuming that those who are most involved in CMR answered to our list, our results are likely to reflect the current best practice of CMR performance.

Lastly, the research covered a period during the COVID-19 pandemic, which might have influenced some of pharmacists’ responses. For example, pharmacists have conducted less CMRs in 2020 than in years prior to the pandemic, or might have experienced lack of time to be a more pronounced barrier due to staff shortages.

## Conclusions

This study showed that community pharmacists in the Netherlands are more efficient in conducting CMRs compared with ten years ago, and some major barriers found back then such as the lack of a systematic approach and suboptimal selection criteria for patients have been overcome. Responding pharmacists believe (jointly) conducting CMRs improves their relationship with GPs and meets patient’s care needs. Lack of time and collaboration with medical specialists were the most important barriers for conducting CMRs. Our study advocates for the involvement of (vocationally educated) pharmacy technicians in the execution of CMRs, as well as the further development and integration of effective ICT solutions to make the process of conducting CMRs more time-efficient. Moreover, future studies should focus on the collaboration between community pharmacists and medical specialists by exploring the perspectives of both roles, in order to gain insight into possibilities to improve collaboration.

### Electronic supplementary material

Below is the link to the electronic supplementary material.

Supplementary Material 1

## Data Availability

Data used in this study is not publicly available.

## Abbreviations

CFIR: Consolidated Framework for Implementation Research
CMR: Clinical Medication Review
CP: Community Pharmacist
Fte: Full-Time Equivalent
GP: General Practitioner
HCP: Healthcare Provider
ICT: Information and Communication Technology
PTAM: Pharmacotherapeutic Audit Meeting
SD: Standard Deviation
