# Supplementary material for: A survey on the implementation of clinical medication reviews in community pharmacies within a multidisciplinary setting
Source: BMC Health Serv Res. 2024 May 3;24:575. doi: 10.1186/s12913-024-11013-z (PMC11067219; doi:10.1186/s12913-024-11013-z)
Supplement: Supplementary file 1 — Supplementary Material 1 [file 12913_2024_11013_MOESM1_ESM.docx]

Appendix A – questionnaire for community pharmacists regarding the implementation of CMRs based on the Consolidated Framework for Implementation Research

Questionnaire for Research: 'Medication Reviews in Public Pharmacies'

Dear Pharmacist,

I would like to invite you to participate in this questionnaire-based research on the implementation of medication reviews in daily pharmacy practice. This research is part of my internship within the Master's in Pharmacy program at Leiden University, under the guidance of Mr. S. Hogervorst MSc and Dr. J.G. Hugtenburg (Amsterdam UMC – VUmc).

Purpose of the Research

The research aims to gain insight into how various pharmacists in the Netherlands carry out medication reviews and the challenges they may experience. We are also interested in understanding the role played by the COVID-19 pandemic in this context. With the results, we hope to provide recommendations that can facilitate the execution of medication reviews in public pharmacies.

Estimated Time to Complete the Questionnaire

Completing the questionnaire will take approximately 15 minutes of your time.

Confidentiality of Data

The data will be used exclusively for this research and will be processed anonymously.

If you have any questions regarding the research, please feel free to contact me at k.kibar@vu.nl.

Best regards,

<Name of researcher>

Medication Review

How many medication reviews were you planning to conduct in 2020?

Number:

How many medication reviews did you conduct in 2020?

Number:

Did you conduct more or fewer medication reviews in 2020 due to COVID-19?

Yes/No*, if yes (next question)

*If 'No' is selected, skip the next question.

Did the COVID-19 pandemic influence the number of planned medication reviews? Please provide details.

Open question:

Which of the following tools and/or criteria do you use when conducting a medication review? Multiple answers are possible.

A. START/STOP criteria

B. PROMISE questionnaire

C. STRIP methodology

D. STOP frail

E. Beers criteria

F. Ephor website

G. Multidisciplinary guideline on polypharmacy

H. Guideline database from the Federation of Medical Specialists

I. NHG standards

J. Medication review guideline from KNMP

K. LESA Care for vulnerable elderly

L. Other, please specify:

Did you involve an external party (such as ReviewExpert or Medi po Farma) for conducting medication reviews in 2020?

Yes/No, if yes, how many medication reviews were outsourced in 2020?

*Skip the next question if 'No' is selected.

Did you use such external services more frequently due to COVID-19 compared to 2019?

Yes/No

Patient Selection

Who is responsible for selecting patients for a medication review?

A. Pharmacist

B. General practitioner or another treating physician

C. Pharmacist and (general) practitioner together

D. Other, please specify:

Which of the following parameters are considered when selecting patients for a medication review? Multiple answers are possible.

A. Residential location (e.g., nursing home)

B. Kidney function

C. Cognition

D. Fall risk

E. Medication adherence

F. Age

G. Gender

H. Number of chronic medications

I. Health insurance provider

J. General practitioner

K. Recent hospitalization or emergency room visit

L. Other, please specify:

Inviting Patients

Who is responsible for inviting patients?

A. Pharmacist

B. General practitioner or another treating physician

C. Pharmacy assistant

D. Pharmaceutical consultant

E. Other, please specify:

How are patients most commonly invited?

A. Phone call

B. Email

C. Invitation letter

D. At the pharmacy counter

E. Other, please specify:

How often did patients or their family/caregivers initiate a medication review in 2020?

A. Never

B. Sometimes

C. Regularly

D. Often

Pharmacotherapeutic History

To what extent does the content of the medication review match the patient's care needs?

O Always O Often O Sometimes O Rarely O Never

Who typically conducts the pharmacotherapeutic history for the medication review?

A. Pharmacist

B. General practitioner or another treating physician

C. Pharmacy assistant

D. Pharmaceutical consultant

E. Other, please specify

Is a (structured) questionnaire used during the pharmacotherapeutic history?

(1) O Always O Often O Sometimes O Never

(2) If yes, which one?

*Skip the next question if 'Never' or 'Sometimes' is selected.

*If (1) O Always O Often

How is this history taken with the patient?

A. Patient fills out the questionnaire

B. Patient and pharmacy staff fill out the questionnaire together over the phone

C. Patient and pharmacy staff fill out the questionnaire together during video calls

D. Patient and pharmacy staff fill out the questionnaire together at the pharmacy

*If (1) O Sometimes O Never

How is this history taken with the patient?

A. Over the phone

B. Via video calls

C. In-person discussion at the pharmacy

D. Other, please specify:

The following statements relate to changes in conducting the history due to COVID-19. To what extent do you agree with them?

I have been conducting the history more often over the phone due to COVID-19 Completely agree Agree Neutral Disagree Completely disagree

I have been conducting the history more often via video calls due to COVID-19 Completely agree Agree Neutral Disagree Completely disagree

I have been conducting the history more often in-person at the pharmacy through discussions due to COVID-19 Completely agree Agree Neutral Disagree Completely disagree

Pharmacotherapeutic Analysis

Who typically performs the pharmacotherapeutic analysis in most cases?

A. Pharmacist

B. General practitioner

C. Pharmacist and general practitioner together

D. Medical specialist and general practitioner together

How much time does the pharmacotherapeutic analysis typically take per patient on average?

A. 0-5 minutes

B. 5-10 minutes

C. 10-15 minutes

D. > 15 minutes

Treatment Plan Development

Who typically creates the pharmacotherapeutic treatment plan in most cases?

A. General practitioner

B. Pharmacist

C. Pharmacist and general practitioner together

D. Other, please specify:

How much time does creating a treatment plan typically take?

A. 0-5 minutes

B. 5-10 minutes

C. 10-15 minutes

D. > 15 minutes

Who is primarily responsible for communicating the treatment plan to the patient?

A. Pharmacist

B. General practitioner

C. Pharmacy assistant

D. Pharmaceutical consultant

E. Pharmacy student

How often did you or the general practitioner initiate a lab test as part of a medication review in 2020?

O Always O Often O Sometimes O Rarely O Never

Follow-up and Monitoring

Who primarily conducts the follow-up and monitoring in most cases?

A. General practitioner

B. Pharmacist(s)

C. Pharmacist and general practitioner together

D. Follow-up is not currently performed in practice

E. Other, please specify:

What system do you use to document findings from medication reviews?

A. Medicijnmonitor

B. NControl

C. AIS

D. Other, please specify:

Effects and Implementation of Medication Reviews

The following statements relate to the effects and implementation of medication reviews. To what extent do you agree with them?

A medication review has a positive effect on the quality of pharmacotherapy Completely agree Agree Neutral Disagree Completely disagree

A medication review enhances the personal treatment relationship with the patient Completely agree Agree Neutral Disagree Completely disagree

Conducting medication reviews improves the relationship with general practitioners Completely agree Agree Neutral Disagree Completely disagree

Conducting medication reviews improves the relationship with specialists Completely agree Agree Neutral Disagree Completely disagree

A medication review has a positive effect on medication adherence Completely agree Agree Neutral Disagree Completely disagree

Medication reviews involving specialists as prescribers are challenging Completely agree Agree Neutral Disagree Completely disagree

A pharmacy assistant can assist the pharmacist in conducting a medication review Completely agree Agree Neutral Disagree Completely disagree

A pharmaceutical consultant can assist the pharmacist in conducting a medication review Completely agree Agree Neutral Disagree Completely disagree

I have insufficient time for conducting medication reviews Completely agree Agree Neutral Disagree Completely disagree

The pharmacist is free to choose the answer options. If left blank, you can proceed to the remaining questions.

Patient-Centered Approach

Which topics are covered in conversations with patients?

Concerns about side effects are discussed

O Always O Often O Sometimes O Rarely O Never

Concerns about the (long-term) effects of medication are discussed

O Always O Often O Sometimes O Rarely O Never

Inability to take medication is discussed

O Always O Often O Sometimes O Rarely O Never

Forgetting to take medication is discussed

O Always O Often O Sometimes O Rarely O Never

Not using prescribed medication is discussed

O Always O Often O Sometimes O Rarely O Never

(Un)intentional medication skipping is discussed

O Always O Often O Sometimes O Rarely O Never

The cost of medication is discussed

O Always O Often O Sometimes O Rarely O Never

Concerns about the quantity of medication are discussed

O Always O Often O Sometimes O Rarely O Never

The availability of medication/alternative packaging is discussed

O Always O Often O Sometimes O Rarely O Never

Other, please specify:

The pharmacist is free to choose the answer options. If left blank, you can proceed to the remaining questions.

Multidisciplinary Collaboration

How often do you consult with a specialist regarding a medication review?

O Always O Often O Sometimes O Rarely O Never

How often do you consult with a home care organization regarding a medication review?

O Always O Often O Sometimes O Rarely O Never

Pharmacy Resources and Facilities

The following statements pertain to resources and facilities that can facilitate or support medication reviews. Please indicate your level of agreement.

The reimbursement received by the pharmacy for a medication review is sufficient Completely agree Agree Neutral Disagree Completely disagree

Health insurers should maintain a (cost-covering) uniform tariff for conducting medication reviews Completely agree Agree Neutral Disagree Completely disagree

Patients are reluctant to participate in medication reviews because the costs are charged against their own risk deductible Completely agree Agree Neutral Disagree Completely disagree

The pharmacy has suitable space for conducting medication review discussions Completely agree Agree Neutral Disagree Completely disagree

The pharmacy's information system adequately supports the execution of medication reviews Completely agree Agree Neutral Disagree Completely disagree

Information systems of different healthcare providers (nursing homes, hospitals, general practitioners, pharmacies) should be compatible Completely agree Agree Neutral Disagree Completely disagree

The pharmacy has sufficient staff hours available for conducting medication reviews Completely agree Agree Neutral Disagree Completely disagree

The pharmacist is free to choose the answer options. If left blank, you can proceed to the remaining questions.

General Characteristics of the Pharmacy

Is the pharmacy part of a chain or franchise?

A. Not part of a chain or franchise.

B. Acdapha

C. Alphega

D. Boots

E. Medsen

F. Pluriplus

G. Service Apotheken

H. Benu

I. Other, please specify:

Pharmacy Patients

How many patients are registered with your pharmacy?

A. 0 - 2500

B. 2500 - 5000

C. 5000 - 7500

D. 7500 - 10000

E. 10000 or more

What is the average socioeconomic status of your patient population compared to other areas in the Netherlands?

A. Low

B. Average

C. High

What is the main demographic composition of your client population?

A. Migration background (non-Western)

B. Migration background (Western)

C. No migration background

D. Other, please specify:

Pharmacy Staffing

How many full-time equivalents (FTEs) of pharmacy assistants work on average at your pharmacy?

Number:

How many FTE pharmacists work at your pharmacy?

Number:

External Organizations

Is there a specific incentive outside the pharmacy for conducting more medication reviews than the number indicated by the IGJ (Inspectorate for Health and Youth Care)?

(1) Yes/No*

(2) If yes, how many additional reviews resulted from this in 2020?

*Skip the next option if 'No' is selected.

Do external organizations also set specific goals?

(1) Yes/No*

(2) If yes, what do such goals relate to? Multiple answers are possible.

A. Execution for specific target population

B. Execution by specific pharmacy staff

C. Execution related to the number of medication reviews

D. Other, please specify:

*Skip the next option if 'No' is selected.

Apart from the goals imposed by external organizations (including the IGJ), have the pharmacy set its own goals?

(1) Yes/No*

(2) If yes, what goal(s)?

*Skip the next option if 'No' is selected.
